# Toward Assessing the Functional Connectivity of Spinal Neurons

**DOI:** 10.3389/fncir.2022.839521

**Published:** 2022-03-03

**Authors:** Martin Zaback, Ekta Tiwari, Alexander J. Krupka, Francesca Marchionne, Francesco Negro, Michel A. Lemay, Christopher K. Thompson

**Affiliations:** ^1^Department of Health and Rehabilitation Sciences, College of Public Health, Temple University, Philadelphia, PA, United States; ^2^Department of Bioengineering, College of Engineering, Temple University, Philadelphia, PA, United States; ^3^School of Engineering, Brown University, Providence, RI, United States; ^4^Department of Biology, DeSales University, Center Valley, PA, United States; ^5^Department of Clinical and Experimental Sciences, Universita degli Studi di Brescia, Brescia, Italy; ^6^Shriner’s Hospital for Children, Philadelphia, PA, United States

**Keywords:** interneuron, motoneuron, high density arrays, single units, spinal cord circuitry

## Abstract

Spinal interneurons play a critical role in motor output. A given interneuron may receive convergent input from several different sensory modalities and descending centers and relay this information to just as many targets. Therefore, there is a critical need to quantify populations of spinal interneurons simultaneously. Here, we quantify the functional connectivity of spinal neurons through the concurrent recording of populations of lumbar interneurons and hindlimb motor units in the *in vivo* cat model during activation of either the ipsilateral sural nerve or contralateral tibial nerve. Two microelectrode arrays were placed into lamina VII, one at L3 and a second at L6/7, while an electrode array was placed on the surface of the exposed muscle. Stimulation of tibial and sural nerves elicited similar changes in the discharge rate of both interneurons and motor units. However, these same neurons showed highly significant differences in prevalence and magnitude of correlated activity underlying these two forms of afferent drive. Activation of the ipsilateral sural nerve resulted in highly correlated activity, particularly at the caudal array. In contrast, the contralateral tibial nerve resulted in less, but more widespread correlated activity at both arrays. These data suggest that the ipsilateral sural nerve has dense projections onto caudal lumbar spinal neurons, while contralateral tibial nerve has a sparse pattern of projections.

## Introduction

Long range monosynaptic projections to spinal motoneurons are relatively rare in the mammalian motor system. Descending projections primarily terminate onto spinal interneurons in order to activate the spinal motoneurons—the classic exception to this are the cortical projections to motor pools which control distal muscles in phylogenetically advanced species (Lemon and Griffiths, 2005). Additionally, large diameter Ia afferents, which are exquisitely sensitive to vibration, have monosynaptic projections to nearly the entire homonymous motor pool (Mendell and Henneman, 1968). However, these specific cortical and reflex pathways are the minority of synaptic contacts on the spinal motoneuron and represent the exception, rather than the rule. Therefore, most synaptic contacts on the spinal motoneuron come from spinal interneurons.

Since the initial work of Lundberg, spinal interneurons have been shown to have a striking convergence across sensory modalities (Hultborn et al., 1976; Jankowska et al., 1981; Kniffki et al., 1981). In addition to this sensory convergence, spinal interneurons also receive and integrate descending drive (Brownstone and Bui, 2010). Thus, interneurons represent a “common path” integrating information from a wide range of sources (Jankowska and Lundberg, 1981). Further, these interneurons are sensitive to neuromodulation from brainstem centers (Schmidt and Jordan, 2000; Husch et al., 2015). This fundamental convergence of inputs and divergence of projections has the potential for high levels of functional connectivity across spinal neurons.

Given the complexities of this system, single or even paired spinal neuron recordings are likely insufficient to fully describe the function of spinal circuits. Spinal microelectrode arrays have been used to record the discharge of populations of individual spinal interneurons in the motor system. This work has been done in several species during a relatively small range of behaviors. Most of these have focused on quantifying interneuronal activity during endogenous behaviors, such as locomotion in the cat (AuYong et al., 2011a,b; Dominguez-Rodriguez et al., 2020; Musienko et al., 2020; McMahon et al., 2021), scratching in the turtle (Radosevic et al., 2019), or under anesthesia in the rat (McPherson and Bandres, 2021). While such behaviors are critical to understand input-output properties within and between interneurons and motoneurons, these relationships are difficult to extract in an intrinsically oscillating circuit. An approach using specific afferent inputs and combining recordings of interneurons and motoneurons would provide a neuronal ensemble view of the relationship between sensory feedback, interneuronal systems, and motor output.

Here, we describe the discharge of spinal interneurons and hindlimb motor units in response to specific forms of afferent drive. Neural recordings from high-density microelectrode arrays in the spinal cord and high-density electrodes arrays on the muscle surface were collected from three cats and decomposed into the individual discharge times of spinal interneurons and motor units. Our initial goal was to describe the functional connectivity of spinal neurons by quantifying short-term correlations within and between lumbar spinal interneurons and hindlimb motor unit discharge patterns. To accomplish this, two forms of afferent drive known to activate spinal interneurons and motoneurons were used—electrical stimulation of either the ipsilateral sural nerve and/or contralateral tibial nerve. Trains of electrical stimulation across a range of frequencies were used to evoke bouts of tonic motor output. Time and frequency domain correlations were used to examine differences in functional connectivity between spinal neurons. The reflex pathways arising from tibial nerve stimulation are thought to diffusely project to multiple spinal segments on the contralateral cord and involve more synapses before reaching the soleus motor pool compared to the reflex pathways arising from sural nerve stimulation (LaBella et al., 1989; Bannatyne et al., 2006). Since the more direct pathway arising from the ipsilateral sural nerve is more likely to have dense projections onto the soleus motor pool, it was expected that sural nerve stimulation would generate greater correlated activation of both spinal interneurons and motor units.

## Materials and Methods

Three adult female domestic shorthair cats (Liberty Research Inc., Waverly, NY, United States, weight: 2.52–3.56 kg) were used for this study. These animals underwent a terminal experiment to evaluate lumbar interneuronal firing and motoneuronal activity during stimulation of either the right (ipsilateral) sural nerve or left (contralateral) tibial nerve.

Cats were initially injected with atropine (0.05 mg/kg IM) and anesthetized with isoflurane (1.5–3.5% in oxygen) supplied through an endotracheal tube. Heart rate, blood pressure, end-tidal CO_2_, tidal volume, arterial oxyhemoglobin saturation, respiration rate and temperature were monitored and recorded every 15 min. Intravenous fluids enriched with sodium bicarbonate (3.4 g/L) and sucrose (25 g/L) were administered throughout the experiment. Dexamethasone (2 mg/kg, IV) was given prior to surgery in order to reduce spinal swelling during the spinal laminectomy. The laminectomy was performed and the spinal cord was exposed at the lumbar level between the L3 and L7 spinal segments. Bipolar nerve cuffs were implanted around the right sural nerve and left tibial nerve at the level of the calcaneal tendon (Ollivier-Lanvin et al., 2011). One bifilar electrode was implanted into the lateral or medial gastrocnemius (LG, MG) muscle and the muscle activity was used to synchronize acquisition between the interneuronal [Tucker-Davis Technologies Inc., (TDT), Alachua, FL, United States] and motor unit [OT Bioelettronica (OTB), Turin, Italy] recording systems using cross correlation of this common signal. Following laminectomy, animals were transferred to a stereotaxic frame where the spinal vertebrae were securely clamped to the frame. The pia was opened to facilitate electrode insertion. The right soleus was exposed and dissected free of the surrounding muscles to facilitate placement of the high-density surface EMG electrode array (Thompson et al., 2018). Decerebration was then performed in order to discontinue anesthesia, which has been shown to affect the activity of the spinal circuitry (Jinks et al., 2005).

### Extracellular Neural Recordings and Processing

One hour after decerebration, *in vivo* recordings of spinal extracellular signals were conducted using two 64 channels microelectrode arrays (model A8x8-5mm-200-200-177, Neuronexus, Ann Arbor, MI, United States) inserted at the dorsal root entry zone to depths of 3,000 to 3,500 μm into two lumbar segments. The planar 8 shaft arrays were inserted sagittally (i.e., in the rostrocaudal direction), so that the recording sites covered a range of 1,450 μm rostrocaudally and 1,450 μm dorsoventrally for each array The rostral electrode array was placed at L3, while the caudal array was placed at either L6 or L7. Interneuronal activity was recorded using the RZ2/RS4 TDT system for recording of 128 channels of multiunit activity (MUA) (sampling rate 24 KHz) and 2 analog channels of EMGs activity (LG/MG) and nerve stimuli (sampling rate 12 KHz). Muscle activity was evoked in the ankle extensors using electrical stimulation of the right (ipsilateral) sural or left (contralateral) tibial nerve. Stimuli were pulse trains (biphasic 100 μs pulses) delivered continuously at one of five possible frequencies (5, 10, 20, 50, and 100 Hz). During each trial, two blocks of stimulation at the same frequency were delivered for 10–20 s with 10 s of rest between. The amplitude of stimulation was adjusted to the minimum current sufficient to evoke robust activity of the ankle extensors (range: 0.2–3 mA for the tibial nerve, and 0.1–1.5 mA for the sural nerve).

**FIGURE 1:**
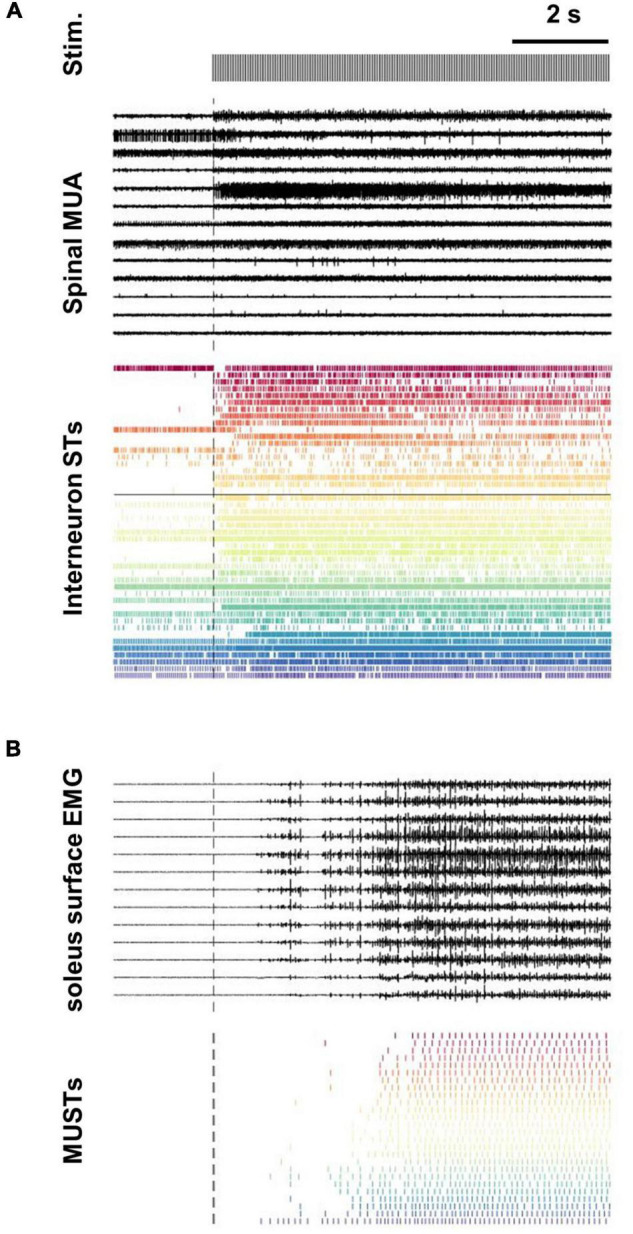
Decomposition of spinal multi-unit and soleus surface electrode array recordings into interneuron and motor unit spike trains. **(A)** Subset of extracellular recordings (13/128 channels) from L3 and L6 spinal intermediate zones are shown along with the population of all interneurons spike trains (STs) decomposed from the array data (*n* = 46). The black horizontal line transecting the interneuron STs separates units decomposed from the L3 (*n* = 27) and L6 (*n* = 19) arrays. **(B)** Subset of surface EMG recordings (13/64 channels) along with the population of motor unit spike trains (MUSTs) decomposed from these array data (*n* = 26).

Neuronal extracellular activity was processed with customized Matlab scripts (The Mathworks, Natick, MA, United States). Raw multiunit data from the RS4 was first band-pass filtered between 300 Hz and 4,000 Hz (sampling rate 24 KHz). Filtered data were then processed using the UltraMegaSort2000 Matlab toolkit (Hill et al., 2011). The units chosen had an average firing frequency greater than 1 Hz throughout the trial, a number of refractory period violations (RPVs) less than 5% for a refractory period of 1.5 ms, and a signal to noise ratio greater than 1.5 (Joshua et al., 2007). Figure 1A depicts a subset of extracellular neural recordings (13/128) from one trial along with all decomposed interneuron spike trains that met the inclusion criteria for subsequent analysis.

### Electromyographic Recording and Processing

Electromyographic (EMG) activity of the soleus muscles were recorded using a 64 channel electrode array placed on the surface of the exposed muscle (Thompson et al., 2018). EMG data were filtered at 20–2,000 Hz, amplified at 150×, and digitized at 5.12 KHz using the Quattrocento system from OTB.

Decomposition of the EMG signal was performed using the well validated blind source separation approach (Holobar et al., 2009; Negro et al., 2016). Only spike trains with a silhouette measure >0.85 were used for further analyses (Figure 1B). Previous investigations have demonstrated the validity of this motor unit-decomposition approach during contractions evoked through cutaneous nerves in the cat, with a 96% rate of agreement with concurrent fine wire electrodes placed in the same muscle (Thompson et al., 2018).

### Spike Train Analyses

**FIGURE 2:**
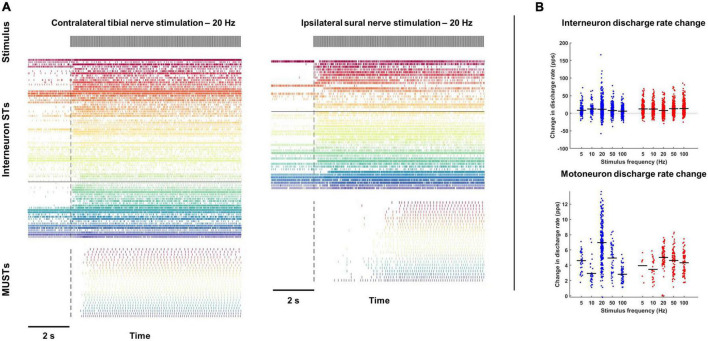
Changes in interneuron and motoneuron spike times during tibial and sural nerve stimulation. **(A)** Population spike times of interneuron and motoneuron single units during exemplar trials of tibial (left) and sural (right) nerve stimulation at 20 Hz. **(B)** With both tibial and sural nerve stimulation, interneurons and motor units changed their discharge rate to a similar degree regardless of stimulation frequency. Motor units were almost exclusively quiescent during the pre-stimulation period and increased their discharge shortly after the onset of stimulation. Most interneurons had some baseline activity and displayed a more heterogeneous response to peripheral nerve stimulation.

Discharge rates of interneuron and motor unit spike trains were calculated during the first block of stimulation as well as during a 2-s period immediately prior to stimulation (Figure 2A). Coefficient variation (CoV) for each motor unit spike train was also calculated during the first block of stimulation after removal of interspike intervals (ISIs) greater than 400 ms.

**FIGURE 3:**
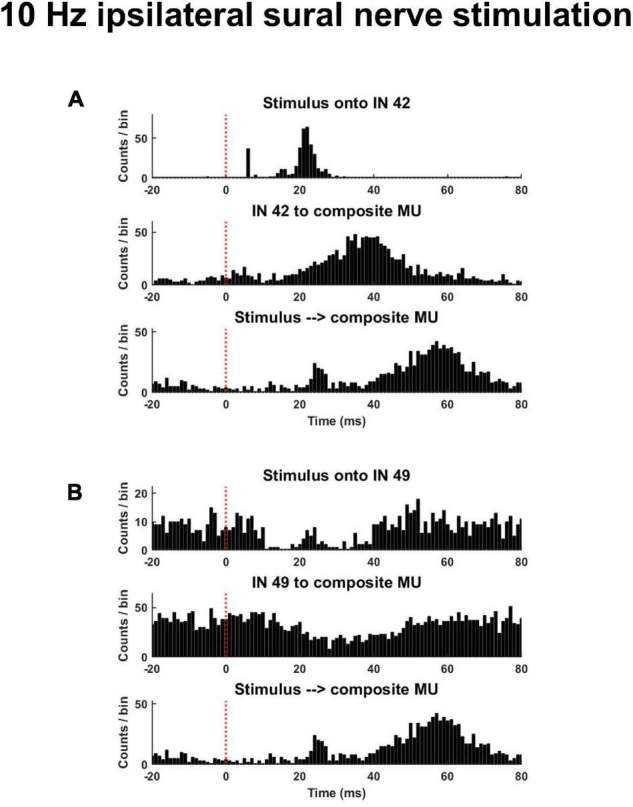
Exemplar peristimulus time histograms during a trial of low frequency (10 Hz) ipsilateral sural nerve stimulation. **(A)** Shown is an interneuron that displayed an increased probability of discharge in response to the stimulus and whose discharge was associated with an increased probability of motor unit discharge [**(A)**, middle panel]. **(B)** Displayed is an interneuron that displayed a reduced probably of discharge and whose discharge was associated with a decreased probably of motor unit discharge [**(B)**, middle panel]. The same composite motor unit spike train in response to the stimulus is shown on the bottom panel of panels **(A,B)**.

**FIGURE 4:**
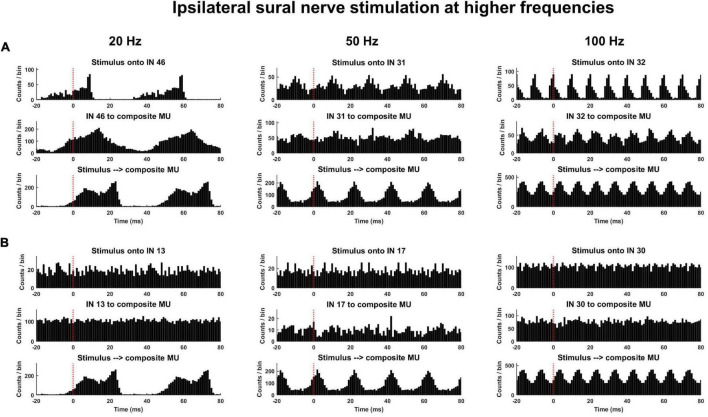
Exemplar peristimulus time histograms during trials of higher frequency (≥20 Hz) ipsilateral sural nerve stimulation. **(A)** Clear responses entrained to the stimulus frequency can be observed; however, due to the long latency of the response and short inter-stimulus interval, the type (excitatory or inhibitory) and onset of response cannot be reliably determined from analysis of the PSTH. **(B)** Illustrates interneurons from the same trial that were not entrained to the stimulus frequency along with composite motor unit spike trains (CST) in response to the stimulus [bottom plots of panels **(A,B)** are repeated].

Time- and frequency-domain correlations were performed to quantify the response of interneurons and motor units to the stimulus as well as the functional connectivity between interneurons and motor units. For time-domain analyses, the following peristimulus histograms (PSTH) were constructed: (1) interneuron spike times aligned to stimulus pulse times (Stim to IN); (2) motor units spike times aligned to stimulus pulse times (Stim to MU); (3) motor unit spike times aligned to interneuron spike times (IN to MU). For PSTH with motor unit spike times (i.e., Stim to MU and IN to MU), all motor unit spike times from a single trial were collapsed into a composite motor unit spike train (CST) (Figure 3). This was done to improve detection of response onsets. For each PSTH, the pre-stimulus period was 20 ms, the post-stimulus period was 80 ms, and the bin widths were 1 ms. To identify the onset and direction of the earliest response for each PSTH, a customized threshold crossing algorithm was used. From the pre-stimulus period, two separate thresholds were calculated: a LOW (±1 SD of mean baseline discharge) and HIGH threshold (±4 SD of mean baseline discharge). The algorithm then searched the post-stimulus period from 5 to 50 ms for any detectable response onset. A detectable response was identified when one of two criteria were met: (1) 6 of 8 consecutive bins exceeded the LOW threshold in the same direction or (2) 2 of 3 consecutive bins exceeded the HIGH threshold in the same direction. These two onset criteria were applied so the algorithm was able to identify small amplitude responses occurring over long durations and large amplitude responses occurring over short durations. The first bin where either of these criteria were met was identified as the response onset. Responses were identified as excitatory or inhibitory based on the direction of change in discharge relative to the pre-stimulus period. These time-domain analyses could only reliably be performed during trials with low frequency stimulation (see Figure 4 and section “Time Domain Correlations Between Stimulus Pulse Train and Interneuron and Motor Unit Spike Trains” below).

The response of interneurons and motor units to the stimulus and connectivity between interneurons and motor units were examined using a coherence analysis based on the methods of Halliday et al. (1995). Coherence reflects linear dependence or correlation between two variables in the frequency domain. To calculate coherence, spike train data were separated into 401 ms disjoint segments (resulting in a frequency resolution of 2.49 Hz). Coherence [*C*_*xy*_(*f*)] was then calculated using equation:


(1) $$\left|C_{xy}\left(f\right)\right|=\frac{{\left|P_{xy}\left(f\right)\right|}^{2}}{P_{xx}\left(f\right)\cdot P_{yy}\left(f\right)}$$

where *P*_*xy*_(*f*) is the averaged cross-power spectral density (PSD) function between the input and output spike trains, and *P*_*xx*_(*f*) and *P*_*yy*_(*f*) are the averaged auto-PSD functions of the same spike trains (Halliday et al., 1995).

**FIGURE 5:**
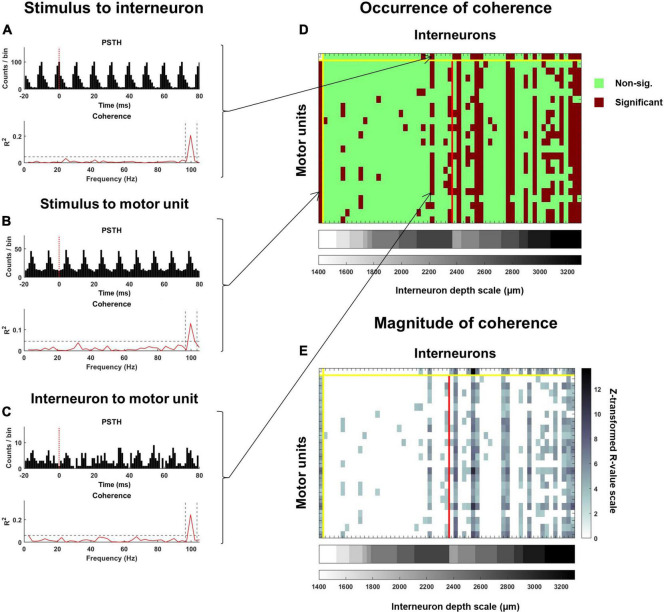
Coherence analysis used to examine the response of interneurons and motoneurons to the stimulus pulse train as well as the functional connectivity between interneurons and motoneurons. Exemplar PSTH and coherence spectra for **(A)** an interneuron spike train relative to the stimulus, **(B)** a motor unit spike train relative to the stimulus, and **(C)** a motor unit spike train relative to an interneuron spike train. On each plot of the coherence spectra, a dashed horizontal line represents a 99% confidence limit. Coherence values exceeding this confidence limit at the stimulation frequency (identified with vertical dashed lines), were considered significant. The occurrence of significant coherence is mapped onto the matrix illustrated in panel **(D)**. The top row indicates interneurons that were and were not significantly cohered to the stimulus pulse times, while this same information for each motor unit is provided along the first column. All subsequent columns starting after the second row indicate instances where an interneuron was significantly cohered with a motor unit. The red vertical line separates interneurons that were recorded at the rostral (left) and caudal (right) spinal segments. Interneurons are further stratified according to depth, with deeper interneurons at each recording site being plotted further to the right. For all significant relations, the peak of coherence (after z-transformation) at the stimulation frequency was calculated. The magnitude of coherence between the stimulus and each interneuron and motor unit, and between each interneuron and motor unit for this exemplar trial is mapped onto the matrix in panel **(E)**.

To identify occurrence of significant coherence between pairs of spike trains, 99% confidence limits were calculated based on the number of segments used to derive the estimate of coherence. When coherence between a pair of spike trains exceeded the confidence limit at the stimulus frequency, they were considered significantly cohered (Figures 5A–C). For all significantly cohered spike trains, the magnitude of coherence was also calculated at the peak of coherence at the stimulus frequency after z-transformation:


(2) $$Z=\left[\text{atanh}\left({\sqrt{C}}_{xy}\left(f\right)\right)/\left(\sqrt{0.5\times L}\right)\right]$$

Where C_xy_ is coherence at the frequency of stimulation (*f*) and L is the number of segments (Figure 5E).

This analysis of coherence was applied to the same combination of variables as the PSTH analyses, except individual motor unit spike trains instead of CSTs were analyzed. Thus, for each interneuron, the percentage of the motor unit population it was cohered to could be calculated (Figure 5D). Similar coherence analyses were performed on pairs of interneurons.

Synchronization between pairs of interneurons was assessed using a PSTH analysis. For these PSTH, the discharge of one interneuron was aligned to the discharge of another interneuron. From a 15 ms pre-stimulus period, 95% confidence limits were constructed and changes in the discharge probability above or below this confidence limit occurring within 1 ms were identified as excitation and inhibition, respectively. This analysis was performed on all combinations of interneurons and the occurrence of excitatory and inhibitory synchrony for each pair of interneurons was calculated.

### Statistical Analyses

All statistical models presented were implemented using SPSS v28 (IBM, Chicago IL, United States). To examine differences in interneuron and motor unit discharge rate as well as motor unit CoV between resting and stimulated states, full factorial linear mixed models of the changes in these variables pre- and post-stimulation onsets with nerve (Tibial vs. Sural) and frequency (5, 10, 20, 50, and 100 Hz) as factors were used (with animal as random factor). Main effects were compared using Bonferroni corrected *post hoc* comparisons between the means, and significant nerve × frequency interactions were followed-up by examining the overlap between the confidence limits of the estimated marginal means at each combination of nerve and frequency.

Linear mixed models were also used to examine how the different afferent drives (ipsilateral sural vs. contralateral tibial) influence the occurrence and magnitude of coherence between the stimulus and motor unit spike trains. Full-factorial linear mixed models with the percentage of significant coherence per trial or the magnitude of coherence for each motor unit significantly cohered with the stimulus as dependent variables, and nerve and frequency as factors (along with animal as a random factor) were conducted. Main effects were compared using Bonferroni corrected *post hoc* comparisons between the means for the factors and significant nerve × frequency interactions were evaluated based on the overlap between the confidence limits of the estimated marginal means at each combination of nerve and frequency.

Linear mixed models were also used to examine how the same afferent drives influence the occurrence and magnitude of coherence between the stimulus and interneurons, and interneurons and motor units. Linear mixed models with the percentage of significant coherence per trial (for Stim to IN) and interneuron (for IN to MU) and magnitude of coherence as dependent variables, and nerve, MUA location, and frequency as factors (along with their 2-way interactions, and animal a as random factor) were conducted to examine the effects of afferent drive on the occurrence and magnitude of these coherences. Main effects were compared using Bonferroni corrected *post hoc* comparisons between the means for the factors, and significant nerve × MUA location interactions were followed-up with an analysis of the effects of nerve and frequency at each MUA location by using a full factorial linear mixed model with nerve and frequency as factors (with animal as random factor) for the variable of interest at each MUA location.

Linear mixed models constructed using the same factors were used to examine how afferent drives influenced the percentage of interneurons time-locked or cohered to each other at each recording location and frequency of stimulation.

To examine the effect of interneuron depth on the occurrence of coherence between interneuron and motor unit spike trains, interneurons were divided into superficial or deep groups using median split based on the depth at which they were recorded. To improve power of these analyses, data were collapsed across stimulus frequency and linear mixed models were conducted separately for each MUA location with fixed effects of nerve and depth and animal as the random effect. For all model interaction and main effects, alpha was set at 0.05.

## Results

### Changes in Interneuron and Motor Unit Firing With Peripheral Nerve Stimulation

Contralateral tibial nerve stimulation responses were measured over 9 trials in animal 1, 10 trials in animal 2, and 7 trials in animal 3, for a total of 26 trials. Ipsilateral sural nerve stimulation responses were measured over 19 trials in animal 1 and 2 trials in animal 2, for a total of 21 trials. From these trials, a total of 2,629 interneuron spike trains were decomposed from the L3 and L6/L7 multi-unit array recordings. Of these interneurons, 81.2% displayed some activity prior to peripheral nerve stimulation, while 67.8% changed their firing in response to stimulation (56.6% increased; 11.2% decreased). This change in firing was observed during both contralateral tibial and ipsilateral sural nerve stimulation (Figure 2A). On average, interneuron firing rate increased during stimulation by 7.1 and 7.9 pps during sural and tibial nerve stimulation, respectively (Figure 2B). There were no significant main effects of nerve (F_1,2448_ = 0.769, *p* = 0.381) or stimulation frequency (F_4,2608_ = 1.759, *p* = 0.134). There was a significant nerve × frequency interaction (F_4,2617_ = 9.023, *p* < 0.001) which appeared to be driven by interneuron firing rate increasing to a greater extent at 20 Hz during tibial compared to sural nerve stimulation. However, *post hoc* examination of the overlap of estimated marginal means 95% confidence limits revealed this effect was not significant.

A total of 678 motor unit spike trains were decomposed from the soleus EMG array data. Motor units were generally quiescent prior to peripheral nerve stimulation (Figure 2A). Only three units displayed some activity during the pre-stimulation baseline period, and all but one unit increased its firing rate during the first block of stimulation. On average, motor units increased their firing rate by 4.8 and 4.7 pps in response to sural and tibial nerve stimulation, respectively (Figure 2B). While there was no significant main effect of nerve (F_1,668_ = 0.160, *p* = 0.689), a frequency main effect was observed (F_4,667_ = 35.072, *p* < 0.001), with 20 and 50 Hz stimulation increasing motor unit firing rate to a greater extent than the other frequencies. There was a significant nerve × frequency interaction (F_4,667_ = 13.54, *p* < 0.001); however, *post hoc* examination of the overlap of estimated marginal means confidence limits revealed no significant effects.

Significant main effects of nerve (F_1,631_ = 73.010, *p* < 0.001) and frequency (F_1,654_ = 30.761, *p* < 0.001) were observed for motor unit CoV. Motor unit CoV was significantly lower during tibial nerve stimulation and was lowest during 20 Hz stimulation during both types of nerve stimulation. A significant nerve × frequency interaction was also observed (F_4,654_ = 11.087, *p* < 0.001). *Post hoc* analysis of estimated marginal means 95% confidence limits revealed this was due to significantly higher CoVs during sural compared to tibial nerve stimulation primarily at lower frequency stimulations (significantly different at 5 Hz).

### Time Domain Correlations Between Stimulus Pulse Train and Interneuron and Motor Unit Spike Trains

Analysis of PSTHs revealed that interneurons displayed a heterogenous pattern of responsiveness to the stimulus pulse train. During low frequency stimulation (≤10 Hz), 36% of interneurons demonstrated an excitatory response to tibial (105/288) and sural (157/434) nerve stimulation. Inhibitory responses were observed in 23% (99/434) of interneurons during sural nerve stimulation and 15% (43/288) during tibial nerve stimulation. While multi-phasic responses could be observed (Figure 3A, top panel), the average onset of the earliest detectable responses were 13.7 ± 9.0 ms to sural stimulation and 14.5 ± 9.0 ms to tibial nerve stimulation.

Longer latency responses were typically observed in motor units (Figure 3, bottom panel). During low frequency stimulation (≤10 Hz), the earliest detectable change in discharge probability of the CSTs were 30.2 ± 12.5 ms and 30.4 ± 12.3 ms during sural and tibial nerve stimulation, respectively. The type of response was not consistent between these modes of stimulation; 100% of CSTs during sural nerve stimulation displayed an excitatory response (6/6), while CSTs during tibial nerve stimulation could display an early inhibitory (3/6) or excitatory (2/6) response.

During low frequency sural nerve stimulation (≤10 Hz), 25.2% (81/322) of CSTs increased their discharge in response to a given interneuron spike train, while 17.3% (56/322) displayed an inhibitory response. This is in contrast to tibial nerve stimulation, during which only 2.1 % (6/288) of CST demonstrated a detectable excitatory response to interneuron discharge, while 2.4% (7/288) displayed an inhibitory response. When responses were observed, their onsets were at similar latencies for sural (23.6 ± 11.6 ms) and tibial (22.1 ± 13.3 ms).

Examination of the timing and type of response of interneurons and motor units to the stimulus pulse train as well as the response of motor units to interneuron spikes were only attempted at lower stimulus frequencies (≤10 Hz). This was because clear phase locking of both interneurons and motor units to the stimulus frequency was observed at higher frequencies (Figure 4). Since response latencies were typically greater than 10 ms, and changes to the probability of discharge could last upward of 40 ms (Figure 3), longer latency responses could fold over into the subsequent stimuli response at higher frequencies (≥20 Hz). This posed challenges for calculating true pre-stimulus firing rates and response latencies using the PSTH approach (Figure 4). The coherence analyses (Figure 5) presented below enabled us to examine relations between stimuli and motor units and interneurons and between interneurons and motor units across all frequencies used in this study.

### Interneuron Coherence With Stimulus Pulse Train

**FIGURE 6:**
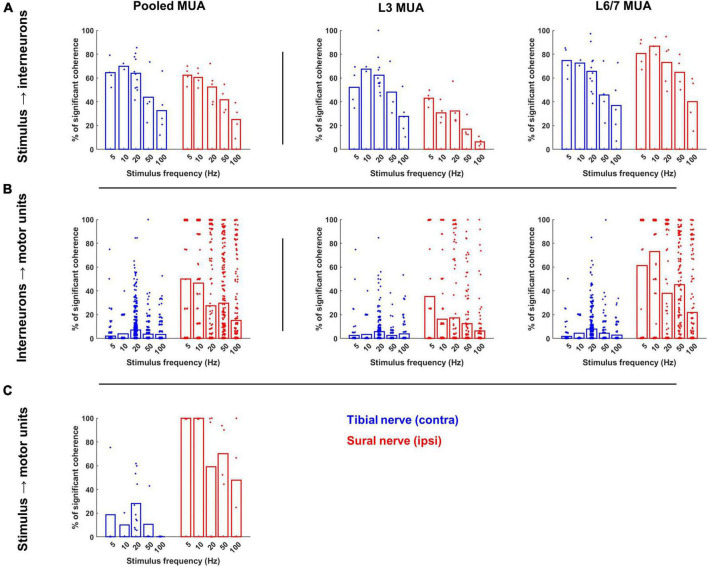
Occurrence of significant coherence during contralateral tibial and ipsilateral sural nerve stimulation across frequencies. **(A)** Percentage of interneurons during each trial that were significantly cohered to the stimulus pulse train. The leftmost plot displays these percentage values for the entire interneuron population recorded during each trial, while the middle and right plots display the percentage of interneurons from each recording site that were cohered to the stimulus. **(B)** Percentage of the motor unit population that were significantly cohered to each interneuron during each trial across the entire population of interneurons (left) and population of interneurons at each recording site (right). **(C)** Percentage of motor pool that was significantly cohered to the stimulus pulse train. Bars represent the average percentage of significant coherence; individual data points have been jittered with uniformly distributed noise to minimize overlap.

During both sural and tibial nerve stimulation, a similar percentage of the interneurons were significantly cohered to the stimulus pulse train (F_1,76_ = 0.310, *p* = 0.579; Figure 6A). Significant main effects of frequency (F_4,75_ = 25.166, *p* < 0.001) and MUA location (F_1,75_ = 90.150, *p* < 0.001) indicated that the percentage of interneurons significantly cohered to the stimulus pulse train decreased as a function of increasing stimulus frequency and were greater at the L6/L7 recording site. A significant nerve × MUA location interaction (F_1,75_ = 39.966, *p* < 0.001) was also observed. Follow-up comparisons of the effect of nerve at each MUA location showed that a significantly greater percentage of interneurons recorded at the L3 site were cohered to the stimulus pulse train during tibial compared to sural nerve stimulation (F_1,35_ = 35.135, *p* < 0.001; Figure 6A middle panel), while the opposite effect was observed for interneurons recorded at the L6/7 site (F_1,36_ = 13.039, *p* < 0.001; Figure 6A right panel).

**FIGURE 7:**
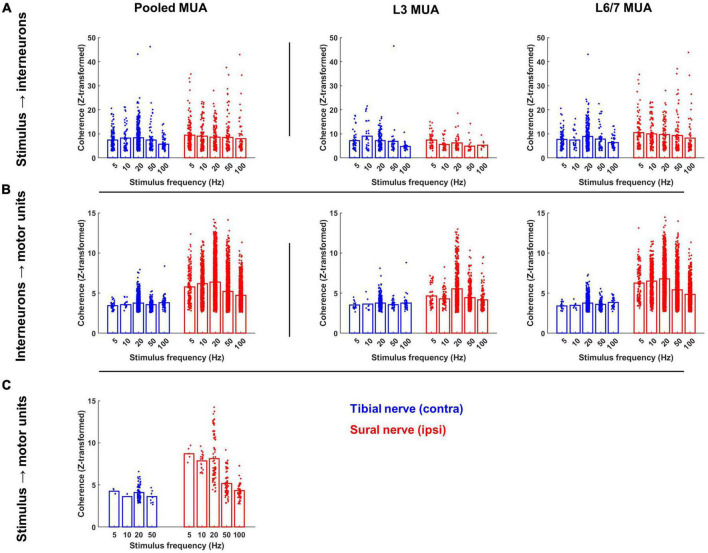
Magnitude of significant coherence during contralateral tibial and ipsilateral sural nerve stimulation across frequencies. **(A)** Peak of coherence between the stimulus pulse train and each interneuron; **(B)** each interneuron and motor unit; and **(C)** the stimulus pulse train and each motor unit at the stimulation frequency. Bars represent the mean coherence value after z-transformation; individual data points have been jittered with uniformly distributed noise to minimize overlap.

A similar pattern of results was observed when the magnitude of coherence between the stimulus and each interneuron was analyzed (Figure 7A). On average, the magnitude of coherence did not significantly differ between sural and tibial nerve stimulation (F_1,437_ = 0.004, *p* = 0.952). However, a significant nerve × MUA location interaction was observed (F_1,1324_ = 19.919, *p* < 0.001). Follow-up comparisons of the effect of nerve at each MUA location showed that the magnitude of coherence recorded at the L3 site was significantly greater during tibial compared to sural nerve stimulation (F_1,179_ = 8.940, *p* = 0.003; Figure 7A middle panel), while the opposite effect was observed for interneurons recorded at the L6/7 site (F_1,156_ = 11.224, *p* = 0.001; Figure 7A right panel). At both MUA locations, there were significant main effects of frequency (L3: F_4,393_ = 2.618, *p* = 0.035; L6/7: F_4,800_ = 3.316, *p* = 0.010) due to coherence generally being weakest at 100 Hz.

### Motor Unit Coherence With Interneuron Spike Trains

When compared to tibial nerve stimulation, a significantly greater proportion of the motor unit population was cohered to the discharge of individual interneurons during sural nerve stimulation (F_1,2355_ = 311.520, *p* < 0.001; Figure 6B left panel). Significant main effects of frequency (F_1,2409_ = 32.131, *p* < 0.001) and MUA location (F_1,2411_ = 221.422, *p* < 0.001) were also observed which resulted from a greater incidence of IN-MN coherence at lower frequencies and at the L6/7 recording site, respectively. A significant nerve × MUA location interaction was also observed (F_1,2411_ = 154.839, *p* < 0.001). Follow-up analyses of the effect of nerve and frequency for each MUA recording site revealed that there was a greater incidence of coherence at both spinal segments during sural nerve stimulation, although this effect was greater at the L6/7 recording site (F_1,1359_ = 605.526, *p* < 0.001) than the L3 recording site (F_1,1049_ = 86.006, *p* < 0.001; Figure 6B, middle and right panels). Significant nerve × frequency interactions were also observed at L3 (F_1,1049_ = 9.758, *p* < 0.001) and L6/7 (F_1,1359_ = 23.946, *p* < 0.001) recording sites. In both cases, *post hoc* analysis of estimated marginal means confidence limits revealed this was due to the incidence of coherence decreasing as a function of stimulation frequency during only sural nerve stimulation.

A similar pattern of results was observed when the magnitude of coherence was analyzed. In particular, a main effect of nerve demonstrated that the magnitude of IN-MN coherence was significantly greater during sural compared to tibial nerve stimulation (F_1,1442_ = 171.292, *p* < 0.001). A main effect of MUA location was also observed due to the magnitude of coherence being significantly greater at the L6/7 recording site (F_1,6122_ = 71.361, *p* < 0.001). A significant nerve × MUA location interaction was also observed (F_1,5508_ = 89.933, *p* < 0.001). Follow-up analyses of the effects of nerve and frequency at each MUA location revealed that the magnitude of coherence was significantly greater during sural compared to tibial nerve stimulation at both MUA locations, although this effect was greater at L6/7 (F_1,4731_ = 193.440, *p* < 0.001) than L3 (F_1,855_ = 29.277, *p* < 0.001). Significant nerve × frequency interactions were also observed at L3 (F_1,1358_ = 6.458, *p* < 0.001) and L6/7 (F_1,4731_ = 15.781, *p* < 0.001) recording sites. *Post hoc* analysis of estimated marginal means confidence limits revealed that these interactions were due to the magnitude of coherence only changing as a function of stimulation frequency during sural nerve stimulation (Figure 7B middle and right panels).

### Motor Unit Coherence With Stimulus Pulse Train

The proportion of the motor unit population significantly cohered to the stimulus pulse train was significantly greater during sural compared to tibial nerve stimulation (F_1,33_ = 23.894, *p* < 0.001; Figure 6C). A significant main effect of stimulation frequency was also observed (F_4,32_ = 3.392, *p* = 0.020), with most follow-up pairwise comparisons indicating that there was a significant reduction in the incidence of coherence during the highest frequency stimulation (100 Hz) independent of the nerve stimulated.

Similar effects were observed when analyzing the magnitude of coherence between the stimulus and motor unit spike trains. The magnitude of coherence was significantly greater during sural compared to tibial nerve stimulation (F_1,283_ = 43.054, *p* < 0.001). A significant main effect of frequency was also observed (F_1,283_ = 30.118, *p* < 0.001); follow-up pairwise comparisons revealed this was due to the magnitude of coherence being significantly lower at frequencies ≥50 Hz. *Post hoc* analysis of a significant nerve × frequency interaction (F_1,283_ = 5.223, *p* = 0.002) indicated that the main effect of frequency was driven primarily by the magnitude of coherence decreasing at higher frequencies during only sural nerve stimulation (Figure 7C).

### Effect of Depth on Incidence of Coherence Between Interneurons and Motor Units

**FIGURE 8:**
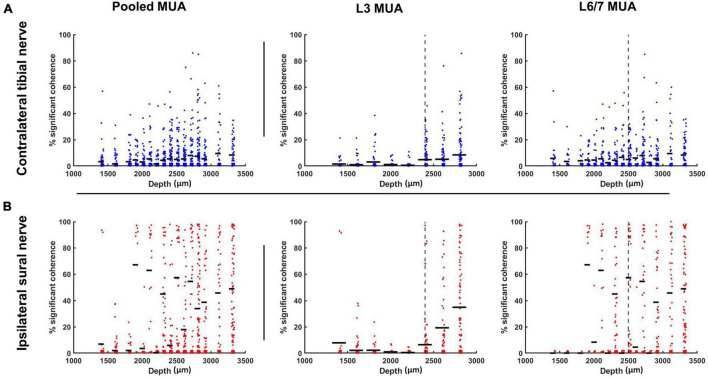
Percentage of motor pool significantly cohered to discharge of individual interneurons (represented as single points) stratified as a function of interneuron depth for contralateral tibial [blue; **(A)**] and ipsilateral sural [red; **(B)**] nerve stimulation collapsed across frequencies. Horizontal dashes represent the mean percentage of motor unit cohered to individual interneurons within each depth bin. The vertical dashed line represents the median depth of the population of interneurons recorded at each spinal recording site. These median values were used to classify interneurons as superficial (≤median) or deep (>median) when examining the effect of interneuron depth on interneuron-motoneuron connectivity.

When a median split was used to classify interneurons as superficial (≤2,400 μm) or deep (>2,400 μm), a significant main effect of depth was observed at the L3 recording site, indicating there was a greater incidence of coherence between motor units and L3 interneurons recorded from more ventral regions of the spinal cord (F_1,952_ = 56.463, *p* < 0.001; Figure 8). However, a significant nerve × depth interaction was also observed (F_1,1051_ = 25.522, *p* < 0.001). While the incidence of significant coherence tended to increase as a function of depth for both nerves, *post hoc* examination of the confidence limits for estimated marginal means revealed that the effect of depth was only significant during sural nerve stimulation.

In contrast to what was observed at the L3 recording site, when a similar median split was applied to interneurons recorded at L6/7 (superficial ≤ 2,500 μm; deep > 2,500 μm), no significant main effect of depth was observed for the occurrence of motor unit coherence with interneurons (F_1,1300_ < 0.001, *p* = 0.985). A significant nerve × depth interaction was observed (F_1,1338_ = 8.866, *p* = 0.003). However, *post hoc* analysis of the overlap of estimated marginal means confidence limits did not reveal any significant effects.

### Coherence Between Interneurons

**FIGURE 9:**
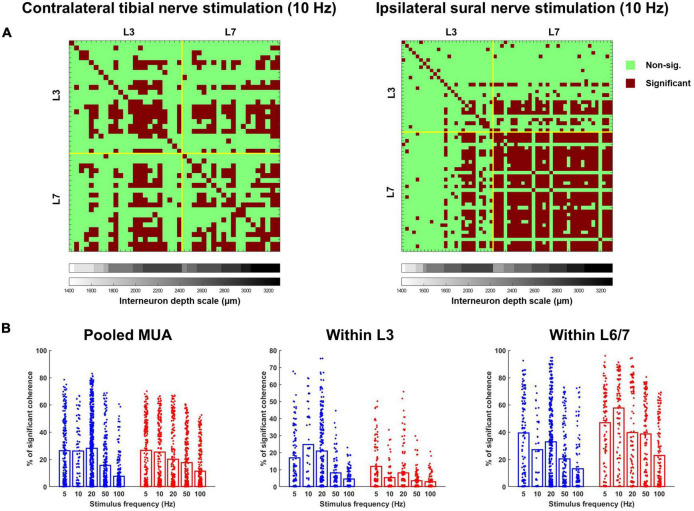
**(A)** Matrices illustrating the incidence of significant coherence between interneurons during exemplar trials of 10 Hz tibial (left) and sural (right) nerve stimulation. Yellow lines separate units recorded at the rostral (L3) and caudal (L6/7) recording sites. Within each recording site, interneurons are stratified by depth, with deeper units plotted further to the right. **(B)** Percentage of interneuron population significantly cohered to discharge of individual interneurons across frequencies in response to contralateral tibial [blue; **(A)**] and ipsilateral sural [red; **(B)**] nerve stimulation. Bars represent means; data points are jittered with uniformly distributed noise to minimize overlap.

All the terms in the model had a significant effect on the percentage of interneurons significantly cohered with each other, with the overall mean for sural stimulation being higher than for tibial stimulation (F_1,2585_ = 55.972, *p* < 0.001) and coherence being twice as high for L6/7 interneurons [mean 37.8 ± 7.4% for L6/7 vs. 15.2 ± 7.4% for L3 (F_1,2585_ = 650.459, *p* < 0.001); Figure 9B]. A significant nerve × frequency interaction was observed for the percentage of interneurons significantly cohered with each other (F_4,2586_ = 3.976, *p* = 0.003). However, *post hoc* analysis of the overlap of the estimated marginal means confidence limits revealed no significant effects. A significant nerve × MUA location interaction was also observed (F_1,2585_ = 187.774, *p* < 0.001). Follow-up analyses of the effect of nerve at the L3 recording site demonstrated that a significantly greater percentage of interneurons were cohered to each other during tibial, compared to sural nerve stimulation (F_1,1071_ = 68.022, *p* < 0.001). By contrast, at the L6/7 recording site, the opposite effect was observed, as a significantly greater percentage of interneurons were cohered to each other during sural nerve stimulation (F_1,1442_ = 180.581, *p* < 0.001). At both MUA locations, significant main effects of frequency were also observed (L3: F_4,1138_ = 41.476, *p* < 0.001; L6/7: F_4,1441_ = 36.141, *p* < 0.001). Examination of pairwise comparisons revealed that in both cases, the percentage of interneurons significantly cohered to each other was significantly lower at higher frequencies (≥50 Hz). Significant nerve × frequency interactions were also observed at the L3 (F_4,1140_ = 12.739, *p* < 0.001) and L6/7 (F_4,1441_ = 3.424, *p* = 0.009) recording sites. At the rostral recording site, *post hoc* analysis of the overlap of estimated marginal means confidence limits revealed that significantly more interneurons were cohered to each other during tibial compared to sural stimulation only at 10 Hz. *Post hoc* analyses at the L6/7 recording site did not reveal any significant effects.

### Zero Lag Synchronization Between Interneurons

**FIGURE 10:**
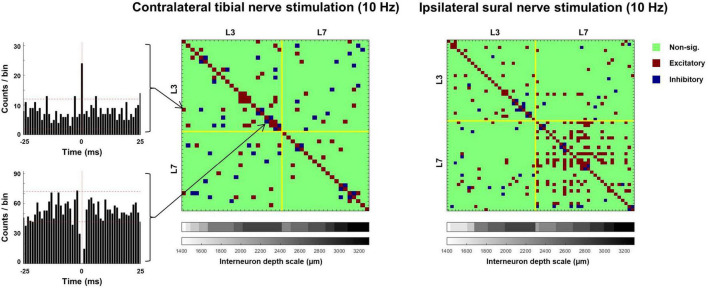
Matrices illustrating the incidence of significant excitatory and inhibitory synchrony between interneurons during exemplar trials of tibial (left) and sural (right) nerve stimulation. PSTHs illustrate exemplar pairs of interneurons displaying excitatory (top) and inhibitory (bottom) synchrony. Dashed horizontal lines on PSTHs represent 95% confidence intervals calculated from a 15 ms pre-stimulus period.

Analysis of interneuron synchronization during 5 and 10 Hz stimulation revealed a significantly greater percentage of excitatory synchronization during sural compared to tibial nerve stimulation (sural: 7.15 ± 4.5%; tibial: 4.60 ± 4.9%; F_1,542_ = 21.407, *p* < 0.001; Figure 10). A significant nerve × MUA location interaction was also observed (F_1,722_ = 46.698, *p* < 0.001). Follow-up analyses revealed that the percentage of interneurons demonstrating synchronous excitation was significantly greater during sural compared to tibial nerve stimulation at the L6/7 recording site (F_1,95_ = 31.716, *p* < 0.001), but not at the L3 recording site (F_1,313_ = 0.108, *p* = 0.743).

Synchronous interneuron inhibition was observed less often than excitation, with 3.90 ± 1.7% and 4.15 ± 1.4% of interneurons displaying this behavior. No terms in the linear mixed model were significant, indicating that the occurrence of synchronous interneuron inhibition did not significantly differ between sural and tibial nerve stimulation (F_1,46_ = 0.457, *p* < 0.502), nor between the rostral and caudal recording sites (F_1,708_ = 0.356, *p* < 0.551).

## Discussion

Recordings from interneurons and motor units were made to quantify the functional connectivity of spinal neurons. Time and frequency domain correlations were performed within and across spinal interneurons and motor units in response to trains of stimulation of the ipsilateral sural or the contralateral tibial nerves. Despite interneuron and motor unit discharge patterns which, overall, were similar across modes of activation, the functional connectivity underlying this activation was different across nerves. In general, our observations are consistent with the hypothesis that the ipsilateral sural nerve has dense projections to the soleus motor pool, while the contralateral tibial nerve has sparse projections to the soleus motor pool.

### Discharge Patterns of Spinal Interneurons and Motor Units

Both sural and tibial stimulation altered interneuron and motor unit discharge rates to similar extents. With both forms of stimulation, interneurons and motor units showed no difference in mean change in discharge rate, although the pattern of discharge was different between interneurons and motor units. A large majority of spinal interneurons were active at rest (McPherson and Bandres, 2021) and showed a heterogenous response to stimulation. Motor units were nearly exclusively quiescent at rest and therefore, necessarily, increased their discharge during stimulation, albeit at half the rate of spinal interneurons. This overall pattern was similar with both forms of afferent drive.

Likewise, the response frequency and latency of interneuronal responses were similar between nerves. Roughly one third of interneurons showed an initial excitatory response to the stimulation, while less than one third showed an initial inhibitory response. The initial response at the level of the motor unit was different between nerves. All the sural responses had an initial excitatory response, while this was more equally split between initial excitatory and inhibitory responses during tibial stimulation. A mixed response is expected given the polysynaptic nature of these pathways (Harrison and Jankowska, 1985; LaBella et al., 1989; Cote and Gossard, 2004; Frigon and Rossignol, 2008). The exclusively excitatory motor unit response during sural nerve stimulation may potentially be explained by a fading of inhibition during trains of stimulation (Heckman et al., 1994).

The latencies of these initial responses were several tens of milliseconds and consistent with previous work on cutaneous reflexes in the cat (Cote and Gossard, 2004; Frigon and Rossignol, 2008). Given such a latency, the afferent pathway responsible for the initial response is likely Aβ fibers, though it is likely that smaller fibers contribute to the later responses. Because of these long latencies, at higher frequencies the response of one stimulation folded over into the response of the next stimulation. This made the analysis of baseline discharge, confidence limits, and response latencies unable to be calculated in a meaningful manner for frequencies greater than 10 Hz. As such, a frequency domain approach was used to better quantify the response across a range of frequencies, however, with these approaches it is difficult to ascertain latencies and if the response is excitatory or inhibitory.

### Differences in Functional Connectivity Between Afferent Pathways

Despite these similarities in change in discharge rate and interneuron response type and latency, stark differences were observed in the correlated activity of spinal neurons during ipsilateral sural and contralateral tibial nerve stimulation. The occurrence and magnitude of coherence was significantly greater for sural stimulation as compared to tibial stimulation. Thus, ipsilateral sural stimulation induced greater entrainment of spinal network activity to the stimulation frequency compared to contralateral tibial stimulation. Further, the occurrence of short latency excitatory synchronous activity among spinal interneurons was greater during sural stimulation than tibial stimulation. Nevertheless, the occurrence of correlated interneuron activity (∼30%) was observed to a greater extent than excitatory and inhibitory synchronous interneuron activity (<10%). This suggests that the network entrainment was more dominant than the putative relatively direct anatomical connections underlying zero latency synchronization.

The location of correlated activity was different between nerves. Sural stimulation evoked greater coherence and excitatory interneuron synchrony in the caudal array, whereas tibial stimulation was no different at L3 as compared to L6/7. While the dorsal root entry for sural and tibial are at or near the L6/L7 spinal level, it is likely that the commissural interneurons needed for the contralateral pathway are more dispersed and have sparse projections to multiple spinal segments (Bannatyne et al., 2006). Consistent with differing projection patterns between the two nerves, these data suggest that the ipsilateral sural nerve has dense projections onto segmental networks of spinal neurons whereas the tibial nerve contralateral pathway, which must synapse onto commissural interneurons since afferents do not cross the midline, show more sparse and spatially distributed projections onto spinal neuron networks (Harrison et al., 1986). Such anatomical organization is consistent with the functional consequence of activation of these pathways—activation of the ipsilateral sural tends to activate ankle extensors, while activation of the contralateral tibial may activate extensors throughout the hindlimb.

The frequency of stimulation had several significant relationships. In general, there is a decrease in correlated activity with increased frequency. This decay is more prevalent in the sural nerve, whereas the tibial nerve has relatively small and uniform coherence across frequencies. As such is it possible that this nerve and frequency interaction plays a critical role the ability of these pathways to transfer information. With its sparse projections, it is difficult for tibial nerve to relay the frequency content of the afferent drive to the soleus motor pool.

### Limitations of the Approach

In the current study, the contralateral tibial and ipsilateral sural nerves were stimulated as means to tonically activate the SOL motor pool (Heckman et al., 1994). This was critical since it was necessary to generate tonic discharge of both interneurons and motor units to examine their connectivity. The methods used in the current study were able to demonstrate clear differences in the pattern of connectivity between these different modes of afferent drive. However, because each of these pathways relay different types of sensory information (i.e., pure cutaneous vs. mixed muscle and cutaneous inputs) from different sides of the body, it is difficult to determine how each of these factors contribute to the differences in connectivity of spinal neurons. Therefore, future studies employing similar methods with a larger sample size should attempt to explore more granular differences in the pattern of connectivity between a variety of afferent pathways.

It is likely that a cutaneous nerve will result in a mixture of inhibition and excitation occurring at different time scales—indeed a subpopulation of interneurons were firing at baseline and decreased their discharge. Our ability to detect such a mixture of excitation and inhibition is limited with the PSTH approach. Because of this, we only characterized the initial response at relatively low frequencies (≤10 Hz). Secondary effects are difficult to observe in the PSTH of motor units due to synchronization of the long after hypolarization of the spinal motoneuron. The after hypolarization in spinal interneurons is likely shorter in duration and it is possible that secondary synaptic events will faithfully be reflected as change in probability of discharge. Peristimulus frequency grams may be used in conjunction with the PSTH approach to better quantify a mixture of excitatory and inhibitory drive at different timescales (Turker and Powers, 2003). Further, recordings from dorsal roots and identification of each interneurons’ preferred activation would be helpful in this regard. This would allow us to gain some measure of the occurrence and latencies of afferent projections to the spinal cord and allow for classification of interneurons. Such information would allow us to more accurately model the neuronal circuitry of the spinal cord.

Additionally, we used the CST to quantify the motor unit response rather than individual motor units. As motor units discharge at much lower rates than spinal interneurons, using the CST increased the number of occurrences and allowed for detection of events. While visual observation of the individuals motor units suggested a homogenous, though noisy, response across the motor pool, recording for longer periods and tracking the same unit would help identify potential non-uniform distributions of synaptic input to the motor pool. Further, as the general change discharge rate was similar between nerves, but the functional connectivity was substantially different between nerves, detailed assessments of individual motoneurons may allow for the detection of features of the state of interneurons using motor unit discharge patterns. Being able to use these motor unit discharge patterns to infer the state of the spinal cord circuitry could provide an important means to non-invasively quantify the state of spinal cord circuitry in humans.

In the current study, estimates of coherence were derived from segments of the data that were weighted equally over the entire duration of the stimulation period. However, it is unclear if the strength of coherence was consistent over time. Repetitive stimulation may cause changes in input-output properties of spinal neurons lasting several tens of seconds due to several factors, including neurotransmitter depletion (Regehr, 2012), impaired vesicle fusion (Neher and Sakaba, 2008), desensitization of post-synaptic receptors (Xu-Friedman and Regehr, 2004), and/or summation of somatic currents (Powers and Binder, 2000; Cushing et al., 2005). Thus, it is possible there may have been time-dependent changes in the magnitude of coherence between the stimulus pulse train and interneuron and motor unit spike times. Future studies should attempt to apply time-frequency analyses to assess time-dependent changes in coherence during periods of repetitive stimulation.

Here we quantified the discharge patterns of spinal interneurons and motor units using time and frequency domain correlations to quantify the functional connectivity of spinal neurons during trains of afferent drive from two different nerves. Our data support the notion that the ipsilateral sural nerve has dense projections to the soleus motor pool, while contralateral tibial nerve has sparce projections. Understanding the activation patterns of spinal neurons during precisely controlled afferent drive will allow for future investigations to quantify how this circuitry changes following neurological injury, such as spinal cord injury.

## Data Availability Statement

The raw data supporting the conclusions of this article will be made available by the authors, without undue reservation.

## Ethics Statement

The animal study was reviewed and approved by Temple University Institutional Animal Care and Use Committee.

## Author Contributions

ML and CT: conception and experimental design. AK, FM, ML, and CT: data collection. MZ, ET, FN, ML, and CT: data analysis. MZ and CT: preparation of figures. MZ, ML, and CT: interpretation of results, manuscript preparation, and critical revisions. All authors contributed to the article and approved the submitted version.

## Conflict of Interest

The authors declare that the research was conducted in the absence of any commercial or financial relationships that could be construed as a potential conflict of interest. The handling editor declared a past collaboration with one of the author ML.

## Publisher’s Note

All claims expressed in this article are solely those of the authors and do not necessarily represent those of their affiliated organizations, or those of the publisher, the editors and the reviewers. Any product that may be evaluated in this article, or claim that may be made by its manufacturer, is not guaranteed or endorsed by the publisher.
